# Impact of Toxigenic *Clostridium difficile* Colonization and Infection among Hospitalized Adults at a District Hospital in Southern Taiwan

**DOI:** 10.1371/journal.pone.0042415

**Published:** 2012-08-02

**Authors:** Yuan-Pin Hung, Pei-Jane Tsai, Kuei-Hsiang Hung, Hsiu-Chuan Liu, Chih-I Lee, Hsiao-Ju Lin, Yi-Hui Wu, Jiunn-Jong Wu, Wen-Chien Ko

**Affiliations:** 1 Department of Internal Medicine, Tainan Hospital, Department of Health, Executive Yuan, Tainan, Taiwan; 2 Department of Experiment and Diagnosis, Tainan Hospital, Department of Health, Executive Yuan, Tainan, Taiwan; 3 Department of Internal Medicine, National Cheng Kung University Hospital, Tainan, Taiwan; 4 Graduate Institute of Clinical Medicine, National Health Research Institutes, Tainan, Taiwan; 5 Department of Medical Laboratory Science and Biotechnology, National Cheng Kung University, Medical College, Tainan, Taiwan; 6 Center for Infection Control, National Cheng Kung University Hospital, Tainan, Taiwan; 7 Department of Medicine, National Cheng Kung University Medical College, Tainan, Taiwan; Charité, Campus Benjamin Franklin, Germany

## Abstract

**Background:**

The impact of toxigenic *Clostridium difficile* colonization (tCDC) in hospitalized patients is not clear.

**Aim:**

To study the significance of tCDC in hospitalized patients.

**Methods:**

A prospective study in the medical wards of a regional hospital was performed from January to June 2011. Fecal samples collected from patients at the time of admission were tested for *tcdB* by real-time polymerase chain reaction (PCR) and cultured for *C. difficile*. The patients were followed up weekly or when they developed diarrhea during hospitalization. If *C. difficile* was isolated, *tcdA* and *tcdB* would be tested by multiplex PCR. The primary outcome was the development of *C. difficile-*associated diarrhea (CDAD).

**Findings:**

Of 168 patients enrolled, females predominated (87, 51.8%), and the mean patient age was 75.4 years old. Approximately 70% of the patients were nursing home residents, and one third had a recent hospitalization within the prior three months. Twenty-eight (16.7%) patients had tCDC, including 16 (9.5%) patients with tCDC at the time of admission and 12 (7.2%) with tCDC during the follow-up period. With regard to the medications taken during hospitalization, the patients were more likely to have tCDC if they had received more than one class of antibiotics than if they had received monotherapy (odds ratio [OR] 6.67, 95% confidence interval [CI] 1.41–31.56, *P* = 0.01), particularly if they received a glycopeptide in combination with a cephalosporin or penicillin or a cephalosporin and a carbapenem. More patients with tCDC developed CDAD than those without tCDC (17.9%, 5/28 *vs.* 1.4%, 2/140, *P* = 0.002). Overall 7 (4.2%) of the 168 patients developed CDAD, and crude mortality rate of those with and without tCDC was similar (21.4%, 6/28 *vs.* 19.4%, 27/140, *P* = 0.79).

**Conclusion:**

Recent use of glycopeptides and β-lactam antibiotics is associated with toxigenic *C. difficile* colonization, which is a risk factor for developing *C. difficile*-associated diarrhea.

## Introduction

*Clostridium difficile* is a major cause of nosocomial antibiotic-associated diarrhea, with clinical features ranging from mild diarrhea to pseudomembranous colitis or toxic megacolon and even death. A large-scale outbreak involving a hypervirulent *C. difficile* strain, B1/NAP-1/027, occurred in Quebec, Canada, in 2003 [1]. The incidence of *C. difficile* infections increased thereafter worldwide, and the trend was accompanied by a substantial increase in the disease severity and mortality rate of *C. difficile* infections. The pathogenicity of *C. difficile* is mediated by at least two exotoxins, toxins A and B, and both damage the human colonic mucosa. The toxins are transcribed from *tcdA* (toxin A) and *tcdB* (toxin B). The outbreak in Quebec was associated with the increased production of toxins A and B in the causative *C. difficile* strain. *C. difficile* infection has been increasingly recognized in Taiwan in recent years, particularly among patients in intensive care units [2]. The dissemination of a predominant *C. difficile* clone has been noted in southern and northern Taiwan [3].

Previous investigators reported that approximately two thirds of patients with fecal *C. difficile* colonization will have persistent colonization during follow up [4], [5]. The prevalence of *C. difficile* colonization was estimated to be 4.4–14% at the time of admission to acute care wards and 4.6–20.4% for chronic care wards [6], [7], [8], [9], [10], [11], [12], [13]. However, the epidemiology of *C. difficile* colonization in the Asian population was lacking. In an earlier study, 15% of patients without initial *C. difficile* colonization acquired *C. difficile* during follow up [10]. However, the incidence of asymptomatic *C. difficile* carriage in long-term care facility residents was up to 51% [14]. The relationship between prior fecal *C. difficile* colonization and *C. difficile-*associated diarrhea (CDAD) remains undefined. One study demonstrated that *C. difficile* colonization was an independent risk factor for CDAD [15], but in another study, asymptomatic *C. difficile* colonization was associated with a decreased risk of CDAD [16]. However, the colonizing *C. difficile* isolates in feces were not clearly identified as toxigenic or nontoxigenic in those studies, particularly in adult Asian patients. Therefore, the relationship between toxigenic *C. difficile* colonization (tCDC) and subsequent CDAD remains controversial.

In previous studies, colonization by toxigenic *C. difficile* strains was studied using cultures and cytotoxin neutralization assays, which were time consuming and technique dependent [17]. Real-time polymerase chain reaction (PCR) to detect the *C. difficile* toxin B gene (*tcdB*) has a high sensitivity and specificity for detecting the presence of toxigenic *C. difficile* isolates [17], [18]. Utilizing real-time PCR, we attempted to study the epidemiology, risk factors, and clinical impact of fecal colonization by toxigenic *C. difficile* isolates and subsequent CDAD in hospitalized adults.

## Materials and Methods

A prospective study was conducted in the medical wards of the Tainan Hospital, Department of Health, Executive Yuan, a district hospital in southern Taiwan, from January 2011 to June 2011. The study was approved by the institutional review board of Tainan Hospital, Department of Health, Executive Yuan, and written informed consent was obtained from all patients. The inclusion criteria for eligible patients included individuals aged at least 18 years old who were admitted to the medical wards with a hospital stay of at least 5 days. The exclusion criteria were as follows: patients with a history of *C. difficile* colonization or infection in three months prior to admission; metronidazole or vancomycin therapy within three months of admission; a clinical diagnosis of CDAD at the time of admission; a history of colectomy.

Diarrhea is defined as a change in bowel habits with more than three unformed bowel movements per day for at least 2 days. Information regarding patient status prior to admission, including comorbid conditions or a history of *C. difficile* colonization or infection, was obtained through oral histories. In addition, medications that may predispose patients to a CDI, such as antibiotics or proton-pump inhibitors (PPIs), that were used for at least one day in three months prior to admission were recorded. The epidemiological analysis was based on the first admission of each patient. All prescribed antibacterial agents were recorded by category. The cephalosporin category included the first- (cefazolin), second- (cefuroxime), third- (ceftriaxone, cefotaxime, and ceftazidime), and fourth-generation (cefepime) cephalosporins. Penicillin derivatives (penicillin, oxacillin, and piperacillin) and the combination of a penicillin derivative and a beta-lactamase inhibitor (amoxicillin-clavulanic acid, ampicillin-sulbactam, and piperacillin-tazobactam) were grouped into the penicillin category. The carbapenem category included imipenem-cilastatin, meropenem, and ertapenem, and the glycopeptide category included vancomycin and teicoplanin.

Fecal samples collected at less than 48 hours after admission and every 7 days during hospitalization were tested by the real-time PCR assay BD GeneOhm™ Cdiff (BD Diagnostics, San Diego, CA), which was kindly offered by the manufacturer, to detect the *tcdB* gene and were cultured for *C. difficile*. Stool samples were collected and transported to the laboratory within 6 hours after collection. Stool was frozen below 4°C before processing. The stool culture procedures for *C. difficile* were as follows. Stool samples were treated with an equal volume of absolute alcohol, homogenized by a vortex mixer, inoculated onto a CCFA (cefoxitin cycloserine fructose agar) plate within 1 hour after collection, and incubated anaerobically for 48 hours in the microbiology laboratory at the Tainan Hospital. If *C. difficile* was isolated, *tcdA*, *tcdB* or 16 S rDNA would be tested for using multiplex PCR, as described previously [19]. Ribotyping was performed for the available *C. difficile* isolates to detect ribotype 027. The DNA sequences of the PCR-ribotyping primers were 5′-GTGCGGCTGGATCACCTCCT-3′ (corresponding to bases 1482 to 1501 of the 16 S rRNA gene) and 5′-CCCTGCACCCTTAATAACTTGACC-3′ (bases 1 to 24 of 23 S the rRNA gene) [20], [21]. If the patients developed diarrhea during hospitalization, real-time PCR and stool cultures of fresh fecal samples were repeated.

A case of tCDC was defined as an asymptomatic individual with *tcdB* detected in a fecal sample by real-time PCR, and CDAD was defined as a symptomatic patient with diarrhea and *tcdB* detected in a fecal sample. All patients included in the study were followed up until discharge or death. The primary outcome was the development of CDAD in patients with or without tCDC. The secondary outcomes were the crude mortality rate at 30 days and the hospital stay duration.

Statistical analyses were performed using statistical software (SPSS, version 13.0). Continuous data were expressed as the mean ± standard deviation. For analyses between patients with and without tCDC, the χ^2^ test or Fisher’s test was used for categorical variables, and Student’s *t*-test was used for continuous variables. A two-tailed *P* value of less than 0.05 was considered to be statistically significant.

## Results

During the 6-month study period, a total of 192 patients were eligible for the study, and 24 patients with no stool available during the first 48 hours after admission were excluded. Thus, 168 patients were enrolled in the study. A total of 254 stool samples were available from 168 patients for testing by real-time PCR and stool cultures. Twenty-six (10.2%) samples grew *tcdA^+^/tcdB^+^ C. difficile* isolates, and all yielded a positive real-time PCR result. In 10 (3.9%) fecal samples, no *C. difficile* growth was observed, but a positive real-time PCR result was noted. Negative findings of both tests were present in 207 (81.5%) samples. Of note, 11 (4.3%) stool samples grew non-toxigenic *C. difficile* isolates and were negative by real-time PCR. Among 37 *C. difficile* isolates, none belonged to ribotype 027.

Eighty-one (48.2%) of the 168 patients were male. The mean patient age was 75.4 years, and 117 (69.6%) patients were nursing home residents. The current use of nasogastric tube feeding was noted in 92 (54.8%) patients. Within 3 months prior to admission, recent hospitalization was noted in 56 (33.3%) patients, 59 (35.1%) had been treated with antibiotics, and 15 (8.9%) had been treated with PPIs. The common underlying diseases included hypertension (55.4%), recent stroke (39.9%), and diabetes mellitus (36.3%). Nine patients had underlying solid organ cancer, but none had received chemotherapy or radiotherapy in the 3 months prior to admission.

**Figure 1:**
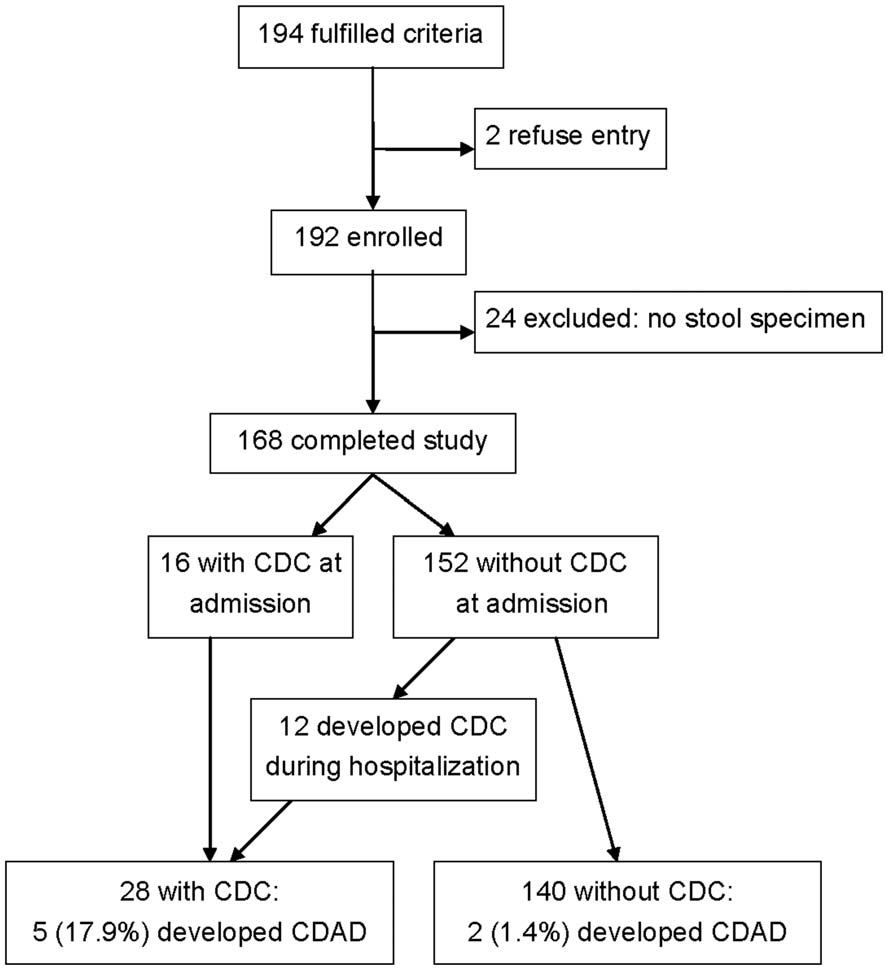
Screening for toxigenic *Clostridium difficile* colonization (tCDC) and subsequent *C. difficile*-associated diarrhea (CDAD).

Of 168 patients, 28 (16.7%) had tCDC, including 16 patients with fecal colonization at the time of admission and 12 who were colonized by toxigenic *C. difficile* during hospitalization (Figure 1). When the 16 patients with tCDC were compared with the 152 without tCDC at the time of admission, tCDC patients had a higher mean body weight (58.1 *vs.* 49.2 kg, respectively, *P* = 0.02) and were often associated with malignancy (18.8 *vs.* 3.9%, respectively, *P* = 0.04) (Table 1). However, there was no difference between the patients with and without malignancy in terms of prior therapy with cephalosporins (33.3 *vs.* 59.1%, respectively, *P* = 0.17), penicillins (33.3 *vs.* 17.6%, respectively, *P* = 0.37), carbapenems (44.4 *vs.* 30.8%, respectively, *P* = 0.47), or glycopeptides (22.2 *vs.* 17.6%, respectively, *P* = 0.66). Additionally, the groups did not statistically differ with respect to hospitalization within 3 months of admission, residence in a nursing home, nasogastric tube use or comorbid conditions. The results of the physical examinations and laboratory findings did not differ between patients with and without tCDC.

**Table 1:** The clinical characteristics of 168 patients with or without toxigenic *Clostridium difficile* colonization (tCDC) at the time of admission.

Characteristics	With tCDC, n = 16	Without tCDC, n = 152	*P* value
Male	7 (43.8)	74 (48.7)	0.80
Age, years	77.8±8.0	75.2±14.9	0.28
Body weight, kg	58.1±11.9	49.2±12.1	0.02
Nursing home resident	11 (68.8)	106 (69.7)	1.00
Recent hospitalization in 3 months prior to admission	8 (50.0)	48 (31.6)	0.17
Nasogastric tube use	8 (50.0)	84 (56.8)	0.61
Antibiotic exposure*	7 (43.8)	60 (39.5)	0.79
Proton pump inhibitor exposure*	1 (6.3)	14 (9.2)	1.00
Underlying medical diseases			
Hypertension	10 (62.5)	83 (54.6)	0.61
Diabetes mellitus	8 (50.0)	53 (34.9)	0.28
Previous stroke	7 (43.8)	60 (39.5)	0.79
Chronic kidney disease (Ccr <60 ml/min)	5 (31.3)	25 (16.4)	0.17
On hemodialysis	1 (6.3)	4 (2.6)	0.40
Malignancy	3 (18.8)	6 (3.9)	0.04

Data are no. (%) of patients, unless otherwise indicated.

* Antibiotic therapy or proton pump inhibitor exposure in the three months prior to admission.

Of the 152 patients without tCDC at admission, 12 (7.9%) developed tCDC in an average of 38.5 (8–88) days after admission. There was no geographic clustering in the units where these 12 patients were located, and no contact with symptomatic patients with CDAD was identified. Nasogastric tube use and comorbid conditions were not associated with the development of tCDC during hospitalization (Table 2). Regarding recent medications, patients acquiring tCDC during follow up were more likely to have prior use of a glycopeptide (odds ratio [OR] 3.63, 95% confidence interval [CI] 1.06–12.45, *P* = 0.05). However, patients who had received more than one class of antibiotics (OR 6.67, 95% CI 1.41–31.56, *P* = 0.01), particularly a glycopeptide plus a cephalosporin (OR 4.50, 95% CI 1.20–16.87, *P* = 0.04) or a penicillin (OR 5.50, 95% CI 1.24–24.38, *P* = 0.04) or a cephalosporin plus a carbapenem (OR 3.63, 95% CI 1.06–12.45, *P* = 0.05), had a higher risk of tCDC. The use of other antibiotics or medications did not result in significantly different risks of tCDC (Table 2). The patients were categorized into four groups by prior exposure to a cephalosporin or penicillin, a glycopeptide, both, or none. tCDC developed in 21.7% of those ever receiving a cephalosporin or penicillin plus a glycopeptide, 6.3% of those with prior use of a cephalosporin or penicillin, and none of those with prior glycopeptide use or without antibiotic exposure (*P* = 0.05). Patients acquiring tCDC during hospitalization were more likely to have CDAD (25.0% *vs.* 1.4%, respectively, *P* = 0.003) and a longer hospitalization (35.0 vs. 20.1 days, respectively, *P* = 0.01) than those without tCDC.

**Figure 2:**
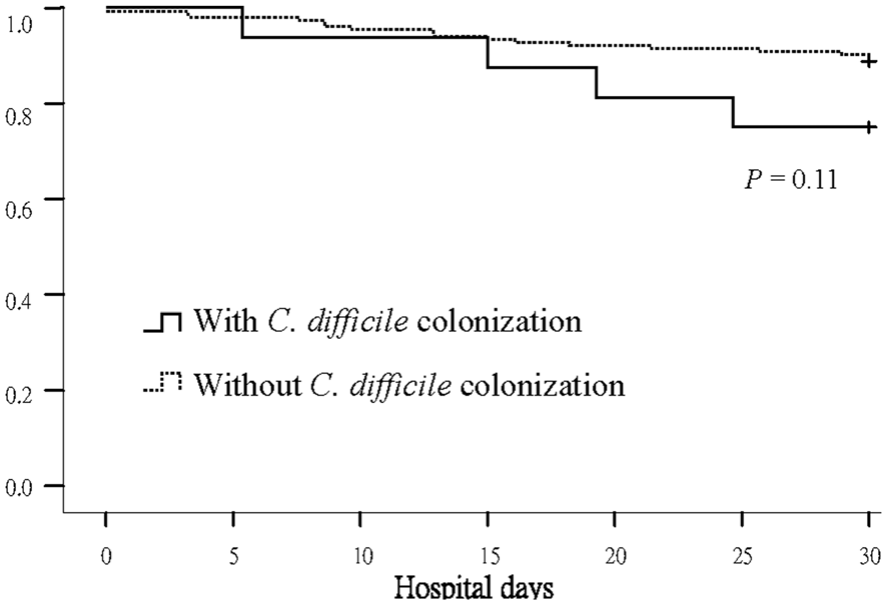
Survival curves of adults with and without toxigenic *Clostridium difficile* colonization at admission.

Overall, 5 (17.9%) of 28 patients with tCDC developed CDAD compared to only 2 (1.4%) of 140 patients without tCDC (*P* = 0.002). Thus, during the 6-month study period, 7 (4.2%) of 168 patients experienced CDAD. The crude mortality rates of those with and without tCDC were similar (21.4%, 6/28 *vs.* 19.4%, 27/140, respectively, *P* = 0.79) (Figure 2).

## Discussion

The prevalence of tCDC, either at the time of admission or during hospitalization, among adults in the medical wards of a Tainan hospital was 16.7%. This is the first epidemiological study of fecal colonization by toxigenic *C. difficile* in Asia. In Hutin’s report in 1997, *C. difficile* colonization was found in 13.3% of patients admitted to infectious disease wards [8], similar to the 16.4% of those admitted to rehabilitation wards in Christina’s report [22]. In Canada, 4.4% of hospitalized patients were found to have asymptomatic *C. difficile* colonization at the time of admission [6]. In another study, 15% of patients without *C. difficile* colonization acquired *C. difficile* during follow up [10]. The prevalence of *C. difficile* colonization may be as high as 20.4% in chronic care geriatric wards [6] and 51% among long-term care facility residents [7], [14]. However, most of these studies were conducted in epidemic settings or high-risk populations. Outbreaks of CDI, particularly toxigenic strains such as ribotype NAP1/027, were reported in the Europe, United States and Canada. However, no recognized outbreak of CDI or the hypervirulent ribotype NAP1/027 strain was identified in Taiwan.

**Table 2:** Prior medications and the risks of toxigenic *Clostridium difficile* colonization during hospitalization in 152 patients without toxigenic *C. difficile* colonization at the time of admission.

Characteristics	Odds ratio (95% confidence interval)	*P* value
Medications taken in the three months prior to admission		
Proton pump inhibitor use	4.79 (1.08–21.19)	0.06
Systemic antibiotic therapy	4.62 (1.14–18.70)	0.04
Medications during hospitalization		
Use of one class of antibiotics		
Cephalosporins	4.00 (0.84–18.81)	0.07
Glycopeptides	3.63 (1.06–12.45)	0.05
Carbapenems	2.33 (0.71–7.65)	0.20
Penicillins	1.76 (0.53–5.82)	0.38
Anti-fungal therapy	3.30 (0.62–17.66)	0.18
Use of ≥1 class of antibiotics		
Any two classes of antibiotics	6.67 (1.41–31.56)	0.01
A β-lactam + glycopeptide		
Cephalosporin + glycopeptide	4.50 (1.20–16.87)	0.04
Penicillin + glycopeptide	5.50 (1.24–24.38)	0.04
Carbapenem + glycopeptide	3.62 (0.98–13.31)	0.06
Cephalosporin + penicillin	6.80 (1.11–41.75)	0.07
Cephalosporin + carbapenem	3.63 (1.06–12.45)	0.05
Penicillin + carbapenem	4.47 (0.80–25.06)	0.12
Other medications		
Corticosteroids	2.09 (0.59–7.46)	0.27
Proton-pump inhibitors	1.20 (0.25–5.89)	0.69

In this study, we found that patients with tCDC were more likely to develop CDAD in accordance with the finding of the study conducted by Lawrence *et al.* in which *C. difficile* colonization was an independent risk factor for *C. difficile* infection [15]. However, the conclusion was contradictory in another investigation, which indicated that asymptomatic colonization with *C. difficile* was associated with a decreased risk of subsequent CDAD [16]. The study was performed in 1998, and the rate of *C. difficile* colonization was evaluated using anaerobic cultures. Real-time PCR has been shown to have a high sensitivity for the detection of *tcdB*-carrying *C. difficile* isolates [17], [18]. Fecal anaerobic cultures had a lower sensitivity for the detection of *C. difficile*, irrespective of toxigenic or nontoxigenic isolates than real-time PCR in our study, as indicated by the finding that 10 (3.9%) stool samples without *C. difficile* growth were positive for *tcdB* by real-time PCR. In contrast, no stool samples yielded toxigenic *C. difficile* and a negative real-time PCR result. Thus, it is generally believed that real-time PCR is an acceptable tool for studying fecal colonization with toxigenic *C. difficile* or CDAD.

The occurrence of CDAD has been associated with prolonged hospitalization [15], [23], [24]. In chronic geriatric wards, the length of hospitalization was longer among patients with culture-confirmed fecal *C. difficile* colonization than that of non-colonized patients (21.6 *vs.* 11.7 days, respectively) [7]. However, these *C. difficile* isolates were not evaluated for toxin production. Currently, little is known about the relative impact of the duration of hospitalization on the risk of subsequent toxigenic *C. difficile* colonization. In our study, the subjects colonized by toxigenic *C. difficile* during hospitalization had a longer hospital stay in the acute care wards. The increased mortality rate associated with CDAD is also of concern [25]. However, our results were not in accordance with such a worrisome notion in western countries. It is likely that the absence of the hypervirulent *C. difficile* clone in Taiwan may have less of a health impact on infected patients.

The relationship between malignancy and CDAD was reported, particularly in patients with hematological malignancy [26], [27] or who were receiving chemotherapy or radiotherapy [28], [29] or a hematopoietic stem cell transplant [30], [31], and was attributed to the immunodeficiency observed in these patients [32], [33]. However, there was no reported interaction between malignancy and tCDC, which was recognized by the univariate analysis in our study. Although prior antimicrobial therapy was not significantly different between individuals with or without malignancy, clinical studies including more cases may be warranted to define the independent clinical factors associated with tCDC.

Almost all antibiotics, including cephalosporins, penicillins, clindamycin, and quinolones, have been associated with CDAD [34], [35], [36], [37]. However, the antibiotics associated with *C. difficile* colonization are unknown. Exposure to clindamycin [8], penicillins [8], or quinolones [14] before admission has been associated with *C. difficile* colonization. Nevertheless, we found that the use of glycopeptides or cephalosporins was associated with *C. difficile* colonization during follow up, although it was not statistically significant. Because prior cephalosporin therapy has been associated with CDAD [36], it is not surprising that we observed an association with *C. difficile* colonization. In contrast, prior glycopeptide treatment was not related to CDAD [36], [38], but in our study, there was a significant association between prior glycopeptide exposure and *C. difficile* colonization. However, the use of parenteral glycopeptide therapy in combination with other antibiotics, such as beta-lactam agents, may be the cause of the discrepancy. The association between *C. difficile* colonization and glycopeptide therapy may be related to glycopeptide-based combination therapy but not the glycopeptide itself. This was demonstrated in our study by categorizing the patients into four groups by prior exposure to β-lactams (cephalosporin or penicillin), glycopeptides, both, or neither; glycopeptide monotherapy was not associated with *C. difficile* colonization. Though the clinical use of more than one class of antibiotics has been associated with CDAD [38], [39], it is not clear which combination regimens are more likely to be associated with CDAD. However, we found that combinations of two classes of antibiotics, especially a β-lactam (either cephalosporin or penicillin) in combination and a glycopeptide, were more often related to *C. difficile* colonization than no antibiotic use.

There are several limitations inherent to our study. First, our case number was small, but this was a prospective cohort studied using a sensitive molecular method to detect toxigenic *C. difficile* colonization or infection. Second, the biological effect of fecal colonization with non-toxigenic *C. difficile*, which most likely will be different from that of fecal toxigenic *C. difficile* colonization, cannot be answered by the present study.

In conclusion, tCDC is a risk factor for developing CDAD, and the combination of glycopeptides and beta-lactam antibiotics was associated with *C. difficile* colonization. These findings may help us to create infection policies and define appropriate antibiotic use in patients with *C. difficile* colonization or CDI.
